# A jaw-dropping complication of Epilepsia Partialis continua following Opercular seizure

**DOI:** 10.1093/omcr/omag170

**Published:** 2026-09-08

**Authors:** Sunil Munakomi, Naphibanroi Lamar, Ishan Shrestha

**Affiliations:** MCh Neurosurgery, Department of Neurosurgery, Birat Medical College and teaching hospital, Tankisinuwari-2, Morang, Biratnagar, Nepal; Junior Resident, Department of Oncology, North Eastern Indira Gandhi Regional Institute of Health and Medical Sciences, Mawdiangdiang, Shillong-793018, Meghalaya, India; Medical Officer, Department of Surgery, Om Hospital and Research Center Pvt. Ltd., Ward no-7, Chabahil, Kathmandu, Nepal

A 42-year-old female underwent decompressive craniectomy and microsurgical clipping for a ruptured middle cerebral artery bifurcation aneurysm with diffuse subarachnoid hemorrhage. On the sixth postoperative day, she developed intermittent paroxysmal involuntary oromandibular movements (Video 1). Urgent CT head demonstrated peri-opercular infarction (Fig. 1). The episodes were clinically suggestive of opercular seizures and subsequently progressed to epilepsia partialis continua (EPC). EEG could not be performed because of the patient’s critical condition and ongoing intensive care management. The diagnosis of EPC was based on continuous rhythmic focal orofacial jerking lasting longer than 60 minutes without impairment of consciousness.

**Figure 1:**
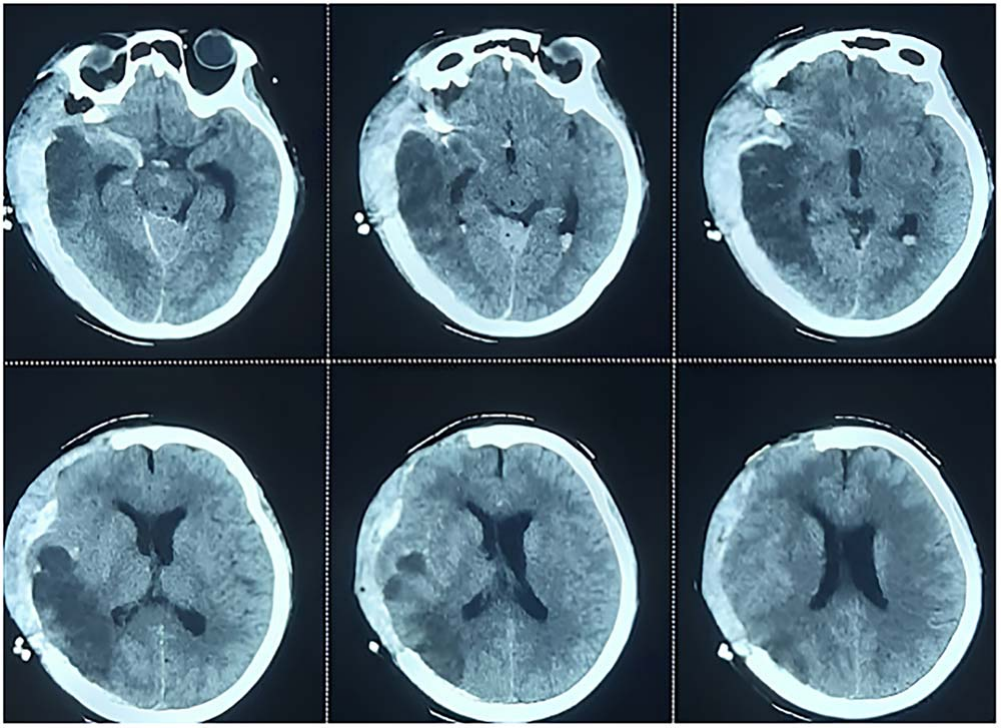
Post-operative CT brain images showing features of right-sided peri-opercular infarction.

Seizures were controlled with intravenous levetiracetam (1500 mg twice daily), intravenous valproate (500 mg twice daily), and lacosamide (200 mg twice daily via nasogastric tube). Following seizure cessation, the patient developed persistent mouth opening, hypersalivation, and jaw dysfunction. Clinical examination suggested bilateral temporomandibular joint (TMJ) dislocation, which was confirmed on CT imaging (Fig. 2).

**Figure 2:**
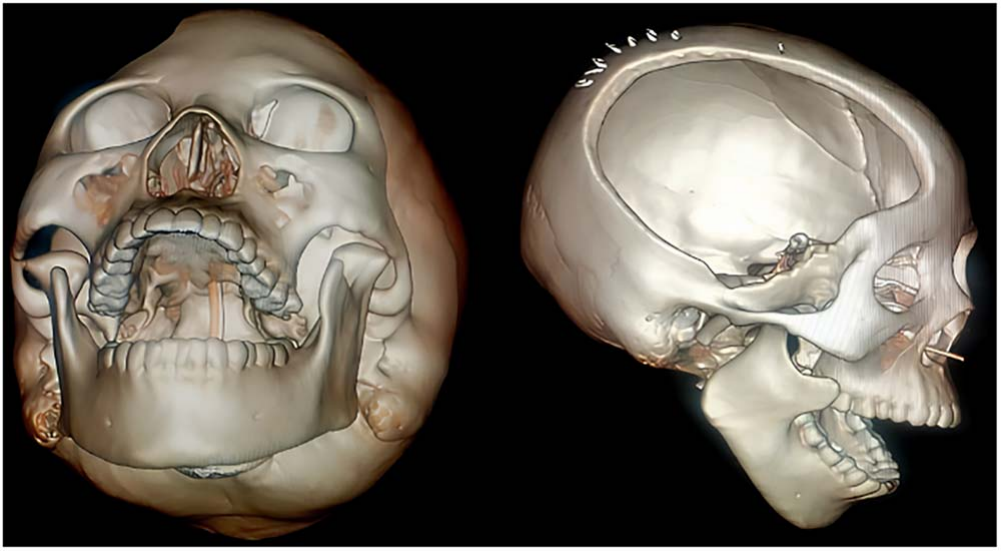
3D CT images showing bilateral anterior temporomandibular joint (TMJ) dislocations in the patient.

Bilateral TMJ dislocation was successfully reduced under intravenous midazolam sedation (2 mg) using Nelaton’s maneuver, with no recurrence during hospitalization.

EPC is a rare form of focal status epilepticus [1]. TMJ dislocation occurring secondary to EPC resulting from opercular seizures remains an under-reported complication [2].

The frontal operculum contains somatotopic representations of the jaw, face, and tongue; seizure activity in this region may cause repetitive contractions of the masticatory muscles. Sustained intense contraction of the lateral pterygoid and masseter muscles can displace the mandibular condyle anterior to the articular eminence, resulting in TMJ dislocation. Even though the seizures originate on one side of the brain (the unilateral operculum), the actual motor output to the jaw usually impacts both sides symmetrically or causes extreme shifting that affects both joints. Moreover, bilateral involvement in our patient can suggest diffuse opercular activation.

Unlike Foix-Chavany-Marie syndrome, which results from bilateral opercular lesions causing loss of voluntary control of facial, lingual, pharyngeal, and masticatory movements, our patient exhibited involuntary seizure-driven oromandibular movements with preserved consciousness, supporting a diagnosis of EPC rather than pseudobulbar dysfunction. TMJ dislocation during seizure activity is uncommon, and its occurrence in opercular EPC is particularly rare [2].

EPC with opercular involvement is itself a rare clinical entity, and its association with bilateral temporomandibular joint dislocation represents an uncommon but clinically significant complication. Early recognition and prompt reduction are essential to prevent aspiration, nutritional compromise, airway-related complications, and prolonged disability [3].

## Supplementary Material

16d0109a-3bd5-4ec7-897b-d2654b13b885_omag170

Video_legend_omag170
