# Supplementary material for: A jaw-dropping complication of Epilepsia Partialis continua following Opercular seizure
Source: Oxf Med Case Reports. 2026 Sep 8;2026(9):omag170. doi: 10.1093/omcr/omag170 (PMC13553001; doi:10.1093/omcr/omag170)
Supplement: Supplementary file 1 [file video_legend_omag170.docx]

Video 1. Involuntary bilateral oromandibular movements suggestive of opercular seizure in the patient.
